# Glycoalkaloid composition explains variation in slug resistance in *Solanum dulcamara*

**DOI:** 10.1007/s00442-018-4064-z

**Published:** 2018-01-30

**Authors:** Onno W. Calf, Heidrun Huber, Janny L. Peters, Alexander Weinhold, Nicole M. van Dam

**Affiliations:** 1 Molecular Interaction Ecology, Institute for Water and Wetland Research (IWWR), Radboud University, Heyendaalseweg 135, 6525 AJ Nijmegen, The Netherlands; 2 Experimental Plant Ecology, Institute for Water and Wetland Research (IWWR), Radboud University, Heyendaalseweg 135, 6525 AJ Nijmegen, The Netherlands; 3 Molecular Plant Physiology, Institute for Water and Wetland Research (IWWR), Radboud University, Heyendaalseweg 135, 6525 AJ Nijmegen, The Netherlands; 4 German Centre for Integrative Biodiversity Research (iDiv) Halle-Jena-Leipzig, Deutscher Platz 5e, 04103 Leipzig, Germany; 5 Institute of Biodiversity, Friedrich Schiller University Jena, Dornburger-Str. 159, 07743 Jena, Germany

**Keywords:** Chemical diversity, Gastropods, Intraspecific variation, HPLC-qToF-MS, Plant–herbivore interaction

## Abstract

In natural environments, plants have to deal with a wide range of different herbivores whose communities vary in time and space. It is believed that the chemical diversity within plant species has mainly arisen from selection pressures exerted by herbivores. So far, the effects of chemical diversity on plant resistance have mostly been assessed for arthropod herbivores. However, also gastropods, such as slugs, can cause extensive damage to plants. Here we investigate to what extent individual *Solanum dulcamara* plants differ in their resistance to slug herbivory and whether this variation can be explained by differences in secondary metabolites. We performed a series of preference assays using the grey field slug (*Deroceras reticulatum*) and *S. dulcamara* accessions from eight geographically distinct populations from the Netherlands. Significant and consistent variation in slug preference was found for individual accessions within and among populations. Metabolomic analyses showed that variation in steroidal glycoalkaloids (GAs) correlated with slug preference; accessions with high GA levels were consistently less damaged by slugs. One, strongly preferred, accession with particularly low GA levels contained high levels of structurally related steroidal compounds. These were conjugated with uronic acid instead of the glycoside moieties common for *Solanum* GAs. Our results illustrate how intraspecific variation in steroidal glycoside profiles affects resistance to slug feeding. This suggests that also slugs should be considered as important drivers in the co-evolution between plants and herbivores.

**Electronic supplementary material:**

The online version of this article (10.1007/s00442-018-4064-z) contains supplementary material, which is available to authorized users.

## Introduction

Plants interact with a large diversity of organisms such as herbivores and pathogens (van Dam 2009). Toxic or deterrent secondary metabolites, such as phenolics, terpenoids and alkaloids are known to govern plant–herbivore interactions (Bennett and Wallsgrove 1994; Howe and Jander 2008). It has been postulated that the large chemical diversity observed in plants today has resulted from the multitude of interactions with herbivores. Each herbivore species may require a specific defence strategy. Generalist herbivores are usually deterred by high levels of secondary metabolites, whereas specialist herbivores have evolved mechanisms to overcome plant defences and may even be attracted by specific secondary metabolites (Ali and Agrawal 2012). However, herbivore communities are not constant in time and space. Therefore, the dominant herbivore species which is exerting the strongest selection pressure on local defence traits may differ among plant populations (Agrawal 2007; Johnson 2011). Since plants evolve specific defence mechanisms against the most damaging herbivore species, differences in dominant herbivore species among plant populations may lead to intraspecific chemotypic variation in secondary metabolite composition (Jones and Firn 1991; Speed et al. 2015).

Slugs and snails are an important component of many herbivore communities in temperate climate zones. These gastropods are widespread generalist herbivores which require moist conditions and intermediate temperatures (Astor et al. 2017; Sternberg 2000). Therefore, slugs are generally more abundant in shaded and moist habitats than in dry habitats with high sunlight exposure. Though often being unnoticed due to their cryptic nocturnal mode of life, gastropods exert a strong selection pressure on natural plant communities and populations (Strauss et al. 2009). Gastropods can affect plant species diversity by selective feeding on seedlings of particular plant species (Barlow et al. 2013; Korell et al. 2016; Strauss et al. 2009). Selective slug feeding may also result in reduced palatability of the surviving plants, as was shown for hybrid willows (Orians et al. 2013). Surprisingly, in the latter example the reduced palatability could not be related to particular differences in defence chemistry, such as phenolic glycosides or tannins (Orians et al. 2013). On the other hand, pine needles with high terpene concentrations as well as artificial diets laced with either one of the terpenes Δ^3^-carene or α-pinene were eaten significantly less by slugs (O’Reilly-Wapstra et al. 2007). Similarly, high-alkaloid accessions of the legume *Lupinus angustifolius* suffered less feeding damage from three different slug species than those with low concentrations of lupin alkaloids (Kozlowski et al. 2017). Together, these studies indicate that gastropods commonly respond to chemical variation within a plant species. Thus they may be an important driver for natural variation in chemical plant defence traits equally to, or even more than, insect herbivores (Gall and Tooker 2017).

The present study focuses on intraspecific variation in gastropod resistance in the bittersweet nightshade, *Solanum dulcamara* (L.). This wild solanaceous perennial woody vine is native to North-Western Europe, Northern Africa and Asia. It is characterized by within and among population genetic and phenotypic variation (D’Agostino et al. 2013; Zhang et al. 2016). High levels of phenotypic plasticity in response to abiotic stress factors allow this species to thrive in habitats with contrasting abiotic conditions, ranging from exposed coastal dunes to wet forest understories (Dawood et al. 2014; Visser et al. 2016). The herbivore community on *S. dulcamara* includes both gastropods and specialist insects (Calf and van Dam 2012; Lortzing et al. 2016; Viswanathan et al. 2005). In a semi-natural Canadian population, taildropper slugs (*Prophysaon sp.*) inflicted up to 15% damage early in the season (Viswanathan et al. 2005). In a German natural population, we found that up to 50% of the plants showed evidence of substantial gastropod feeding (Lortzing et al. 2016). These observations illustrate the importance of slugs as natural herbivores and potential drivers of defence evolution in *S. dulcamara.*

*S. dulcamara* produces steroidal glycoalkaloids (GAs), which are highly toxic and deterrent to many organisms (Eich 2008; Kumar et al. 2009; Milner et al. 2011). Previous studies found that there is (genetically fixed) chemotypic variation in GA profiles among individuals of *S. dulcamara* (Mathé 1970; Willuhn 1966; Willuhn and Kunanake 1970). It is known that alkaloidal secondary metabolites generally deter gastropod feeding (Bog et al. 2017; Speiser et al. 1992; Wink 1984). Thus it is very likely that differences in GA concentrations and profiles also affect resistance to slugs. However, to our knowledge the ecological consequences of *S. dulcamara* GA concentrations or profiles for plant–herbivore interactions have not been investigated so far.

We utilized the naturally available genetic variation within and among populations of *S. dulcamara* to address the following specific questions: (1) Is there intraspecific variation in gastropod resistance in *S. dulcamara*? (2) What are the underlying chemical mechanisms explaining variation in gastropod resistance in *S. dulcamara*? We addressed these questions in a series of bioassays using the grey field slug (GFS, *Deroceras reticulatum* Müller) as a gastropod model species. Although there are no actual data available on its natural hosts, GFS is an abundant generalist gastropod species which occurs sympatrically with *S. dulcamara* (South 1992). Adults are relatively small (3–4 cm), and well suited to be used in high-throughput preference assays on leaf discs in Petri dishes (Hendriks et al. 1999). We hypothesised that intraspecific variation in GFS resistance is related to plant chemical diversity. The preference assays were combined with a metabolomics approach to identify the chemical mechanisms underlying differences in slug preference. This allowed us to link the slug’s choice behaviour directly to variation in the chemical profiles of the different accessions.

## Materials and methods

### Plant material

**Fig. 1:**
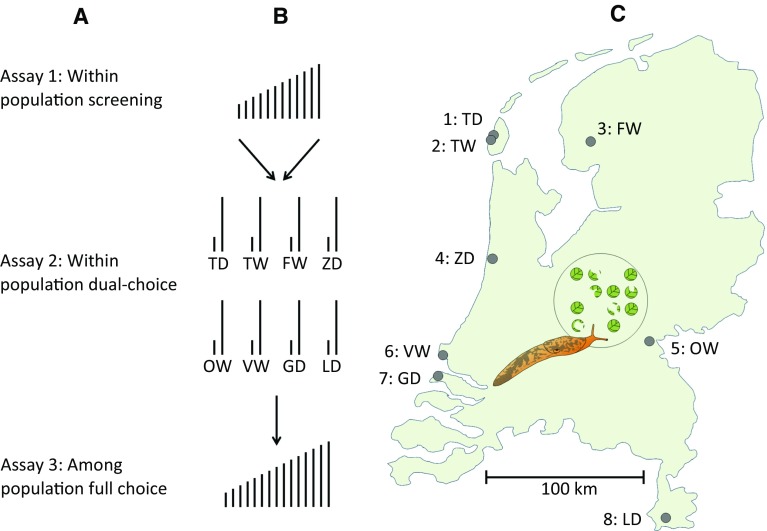
Experimental design of the consecutive assays. Column **a**: short title indicating the aim of the three consecutive slug-preference assays. Column **b**: graphical representation of the accession selection and testing procedures. Column **c**: geographic locations of eight *Solanum dulcamara* populations in the Netherlands. *TD* texel dry, *TW* texel wet,* FW* friesland wet,* ZD* zandvoort dry,* OW* ooijpolder wet,* VW* voorne wet,* GD* goeree dry,* LD* limburg dry

Eight native *S. dulcamara* populations from the Netherlands were selected based on the criterion of covering a wide geographic area and range of abiotic conditions, ranging from dry open coastal dune areas to river floodplains and lake borders (Fig. 1 and ESM Table 1). This selection was made to capture intraspecific variation in local conditions, such as differences in herbivore community composition, which may be a causal agent for selection on plant defence traits. Seed batches of the source populations, which were field collected as in Zhang et al. (2016), were provided by the Radboud University Genebank (http://www.ru.nl/bgard/). Seeds were cold-stratified at 4 °C in the dark on a 2 cm layer of glass beads (1 mm *Ø*) and tap water in a plastic container (8 × 8 × 6 cm,* L* × *W* × *H*, www.der-verpackungs-profi.de GmbH, Göttingen, Germany) covered with a transparent lid for at least 2 weeks. Germination was initiated by transferring the container to greenhouse conditions. After approximately 8 days, when cotyledons had unfolded, seedlings were transplanted into individual pots (11 × 11 × 12 cm,* L* × *W* × *H*) containing potting soil (Lentse Potgrond nr. 4, Horticoop, Katwijk, The Netherlands) supplemented with 4 g L^−1^ slow-release fertilizer (Osmocote^®^ Exact Standard, Everris International B.V., Geldermalsen, The Netherlands). Each seedling, hereafter referred to as ‘accession’, received a unique accession number. This number is composed of a two-letter population abbreviation followed by a number (1–12). To generate sufficient plant material for experimentation, the accessions were propagated by cloning. Stem cuttings, consisting of a single node with at least 2 cm of woody stem internodes on the distal and proximal side, were potted directly in the same soil mixture as above. The soil was kept moist to stimulate adventitious root formation. All plants were grown in net cages within a greenhouse to prevent insect infection (Rovero 0.3 mm gauze, 7.50 × 3 × 2.75 m,* L* × *W* × *H*). Greenhouse conditions were set to maintain a 16-h photoperiod with minimum temperatures of 20 °C/17 °C (day/night). Light levels were supplemented with 1000 W sodium lamps (Philips GreenPower, Amsterdam, The Netherlands) fixed above the net cages providing ~ 280 µmol m^−2^ s^−1^ light intensity at the plant level. Details on the size and age of plants used for experiments are given below.

### Slug material

GFS individuals were frequently collected in the field in the vicinity of Nijmegen (the Netherlands) and individually kept in clear 50 ml plastic containers (6 cm *Ø*, www.der-verpackungs-profi.de GmbH, Göttingen, Germany) lined with sieved (2 mm mesh) humid potting soil. Containers were placed in a climate cabinet (Snijders Scientific, Tilburg, The Netherlands) under 16-h low light photoperiod of ~ 50 µmol m^−2^ s^−1^ light intensity at temperatures set to 17 °C/14 °C (day/night). The diet consisted of self-grown organic lettuce (Bio Pluksla ‘Mesclun’, Dille & Kamille, Zoetermeer, The Netherlands), which was refreshed twice a week. Containers were cleaned every week by removing faeces, diet residues and excess water. Slugs were transferred to clean containers with fresh soil monthly.

### Slug preference assays

A series of three sequential slug-preference assays was performed to investigate natural variation in slug resistance in *S. dulcamara* (assay 1–3 in Fig. 1). In assay 1, accessions (*n* = 12 per population) grown from seeds collected in eight native source populations were screened to assess within population variation in GFS preference. In assay 2, leaves of stem cuttings, hereafter referred to as clones, of the most- and least-preferred accession in each population were offered to GFS in a dual-choice assay to test whether the feeding preference was consistent. In assay 3, leaves of clones of 15 out of the 16 accessions used in assay 2 were offered to GFS in a full-choice preference assay to assess overall preference for accessions among the eight original populations.

### General approach

The same general experimental set-up was used for all three assays. Specific details are given per assay (see below). Plants used for the bioassays were of equal age, between 60 and 100 cm tall and any inflorescences that were emerging were regularly removed. Because the accessions showed variation in growth rates, only fully expanded leaves at a position of about 2/3rd of the stem length measured from the apex were used to ensure that leaves of similar developmental stage and metabolic composition were used in all assays. Leaves were numbered from the apex downwards, starting with the first leaf below the first internode that was greater than 2 cm. Within each assay, leaves from the same position were used for all accessions/clones. Leaf discs were made of interveinal tissue using a cork-borer (1.5 cm *Ø*). One leaf disc of each treatment/accession was placed in a Petri dish (9 cm *Ø*) with the adaxial (i.e. upper) side down. The leaf discs adhered sufficiently strong to the bottom of the Petri dish to prevent them from changing their position due to slug activities. For each Petri dish, leaf positions of the different accessions within a given Petri dish were based on a unique pre-determined and completely randomised order which was printed on paper and placed under the transparent dish when setting up the assay. The Petri dishes were gently sprayed with de-ionised water before, during and after placing the leaf discs to create a moist environment for maintaining leaf disc quality. Depending on the size of the slugs, either two or three individuals were placed on the lid of each Petri dish after which the dishes were closed and placed in the slug-culture cabinet. After 24 h, slugs were removed and the leaf material remaining in the Petri dish was photographed with a 14 cm ruler as scale reference for analyses of consumed area using ImageJ v. 1.48 (Schneider et al. 2012). Each slug was only used once for experimentation.

### Assay 1: eight population screenings

Twelve randomly selected 4- to 5-week old seedlings per source population (*n* = 8) were used for preference assays to assess within-population variation in slug feeding resistance. Each individual was given an accession identifier consisting of the population code (Fig. 1) and a sequential number. Three leaves were selected from each accession (leaves 6–8 from the apex). From each leaf, 8 leaf discs were punched, providing a total of 24 discs (3 leaves × 8 discs) per accession. Individual discs were randomly allocated to Petri dishes (*n* = 16) for preference assays. Each Petri dish thus contained 12 leaf discs, each representing 1 of the 12 accessions of a single population. One accession of Limburg Dry (LD12) was discarded right before the onset of the preference assay due on the suspicion of being infected with a disease, leaving 11 accessions for this source population.

### Assay 2: within population dual-choice

For each of the eight source populations, the most- and least-preferred accessions in assay 1 were selected. As the initially chosen accessions TD11 and FW03 appeared to be infected by a disease, these were replaced by the second most-preferred (FW09) or least-preferred (TD01) accession. Per accession, three 4- to 5-week-old clones with similar stem lengths were selected for a within population dual choice assay to test the consistency of the slugs’ preference between the most and least preferred accession for each source population. From each plant, 1 leaf was selected (leaf 7 from the apex) from which 8 leaf discs were made, thus providing a total of 24 discs (1 leaf × 3 clones × 8 discs). Discs were randomly allocated over Petri dishes (*n* = 8 per population). Each Petri dish received 3 discs of the most-preferred (1 disc of each clone) and 3 of the least preferred (idem) accession, resulting in 6 leaf discs presented to the slugs in each Petri dish.

### Assay 3: among population full-choice

New clones were made from the 16 accessions selected for assay 2. Because accession TD01 appeared diseased and was excluded from further assays, only 15 accessions were used in assay 3. Three 7-week-old clones with similar stem length were selected for each accession. From each plant, 2 leaves were chosen (leaves 10 and 11 from the apex) and 4 leaf discs were made from each leaf, resulting in 24 discs for each accession (2 leaves × 3 clones × 4 discs). The leaf discs were pooled and single discs were randomly allocated to Petri dishes (*n* = 19) for the 15-choice assay. Three replicates of the preference assay were excluded from statistical analyses due to excessively low or high consumption rates or being unable to reconstruct the original leaf disc position, leaving a total of 16 suitable replicates. Four leaf discs of each accession were oven-dried to constant weight and used to determine the specific leaf area (cm^2^ g^−1^ dry weight).

### Statistical analyses of preference assays

Absolute leaf disc consumption data (cm^2^) of all preference assays were analysed using nonparametric statistical methods from the R “stats” package R Core Team (2016). Friedman’s rank sum test was applied to evaluate overall preferences for accessions in the multiple choice assays (assays 1 and 3) using the Petri dish number as grouping factor. Paired Wilcoxon signed-rank tests with continuity correction, excluding ties (“no choice”), were applied to assess differences in preferences for two accessions (assay 2). For presentation in figures, the absolute consumed leaf area was converted to the relative consumption per Petri dish $\left( {\frac{{{\text{Individual leaf disc area consumed}}\; \left( {{\text{cm}}^{2} } \right)}}{{{\text{Total leaf disc area consumed in Petri dish}} \;\left( {{\text{cm}}^{2} } \right)}}} \right)$ to correct for individual differences among slugs, across Petri dishes, and experimental series. A Pearson’s correlation test was performed to test the relation between the relative leaf disc consumption and the specific leaf area in assay 3.

### Metabolic profiling using HPLC-qToF-MS

The leaf tissue immediately surrounding the area of the leaf discs used for assay 3 was dissected at ~ 1 cm circumference around the original hole, collected in screw cap tubes (57.0 × 15.3 mm, Sarstedt AG&Co. Nümbrecht, Germany), flash frozen in liquid nitrogen and stored at − 80 °C until further processing. These small leaf tissue samples taken from three clones per accession were pooled per accession, resulting in 15 leaf samples for metabolomic analyses.

A semi-untargeted analysis with particular emphasis on abundant compounds was performed to determine which chemical compounds relate to slug preference. Leaf samples were extracted following a procedure derived from de Vos et al. (2012). In short, fresh leaf material was ground in liquid nitrogen. About 100 mg of ground sample was double extracted with, respectively 1.0 and 0.9 ml MeOH:acetate buffer (pH 4.8) in 2 ml reaction tubes holding two glass beads (5 mm *Ø*) by shaking in a TissueLyser (Qiagen, Venlo, the Netherlands) at 50 Hz for 5 min followed by centrifugation at 15.000 rpm at 4 °C. Clear supernatants were combined and stored at − 20 °C until further processing.

Two sets of diluted crude extracts (1:5 and 1:50) were analysed with an UltiMate™ 3000 Standard Ultra-High-Pressure Liquid Chromatography system (UHPLC, Thermo Scientific) equipped with an Acclaim^®^ Rapid Separation Liquid Chromatography (RSLC) 120 column (150 × 2.1 mm, particle size 2.2 μm, ThermoFischer Scientific) using the following gradient at a flow rate of 0.4 ml min^−1^: 0–2 min, isocratic 95% A [water/formic acid 99.95/0.05 (v/v %)], 5% B [acetonitrile/formic acid 99.95/0.05 (v/v %)]; 2–15 min, linear from 5 to 40% B; 15–20 min, linear from 40 to 95% B; 20–22 min, isocratic 95% B; 22–25 min, linear from 95 to 5% B; 25–30 min, isocratic 5% B. Compounds were detected with a maXis impact–quadrupole time-of-flight mass spectrometer (qToF-MS, Bruker Daltonics) applying the following conditions in positive ionization mode: scan range 50–1400 m/z; acquisition rate 3 Hz; end plate offset 500 V; capillary voltage 3500 V; nebulizer pressure 2 bar, dry gas 10 L min^−1^, dry temperature 220 °C. Mass calibration was performed using sodium formate clusters (10 mM solution of NaOH in 50/50 (v/v  %) isopropanol water containing 0.2% formic acid).

The 50 most prominent peaks (signal: noise > 10) in the chromatograms of 15 accessions—hereafter referred to as compounds—were selected for further analyses. Their intensities were determined based on the most characteristic fragment and normalised by extracted fresh weight. The mean relative consumption of GFS on leaf discs of the 15 accessions was correlated with the log_10_-transformed peak intensities g^−1^ FW of all 50 compounds using Pearson’s correlation analyses applying correction for the false discovery rate (FDR) using the online R-based tool MetaboAnalyst 3.0 (Xia et al. 2015). Tandem mass spectrometry (MS^2^) spectra were acquired by injection of samples that contained the highest amount of compounds of interest using the same chromatographic conditions as described above. MS^2^ spectra were collected using the automated MSMS function of the Bruker oToF Control software. Spectra were evaluated for compounds of interest with particular emphasis on fragmentation of the parental compound, to understand the structural composition of backbones and possible conjugations. Putative identifications were made based on comparison of mass spectra reported in the literature (Lu et al. 2008; Munafo and Gianfagna 2011; Shakya and Navarre 2008). Solasonine and solamargine were identified by injection of authentic standards (Carbosynth Limited, Berkshire, United Kingdom) and comparison of retention time and mass spectra.

## Results

### Intraspecific variation in slug feeding resistance

**Fig. 2:**
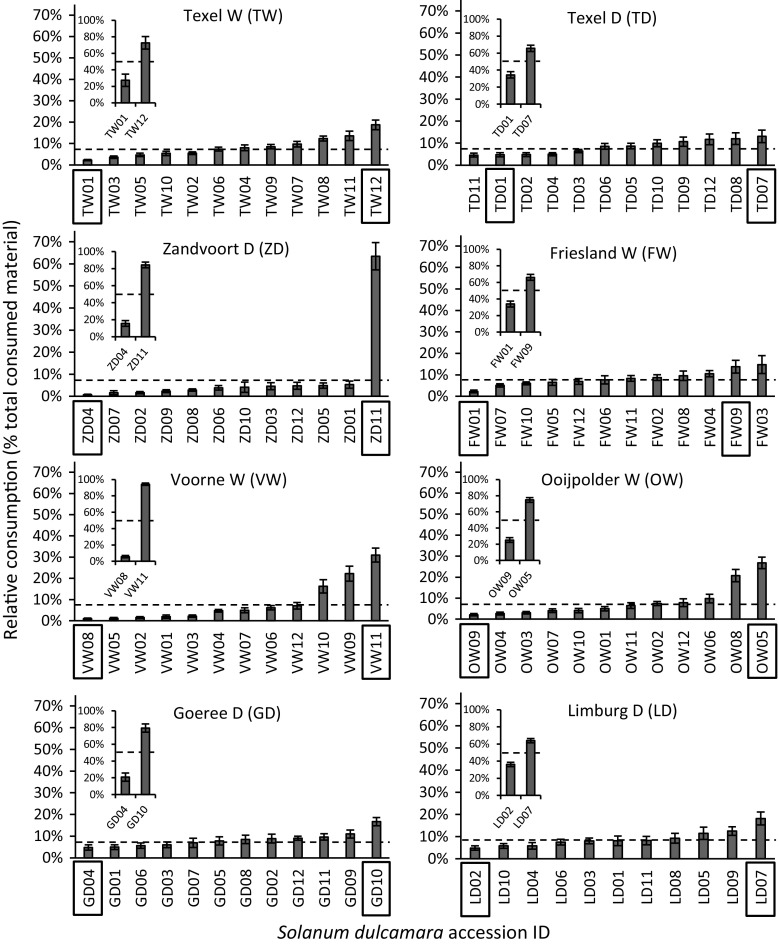
Mean relative consumption (± SE) of grey field slugs in independent preference assays on *Solanum dulcamara* leaf discs. Large panels show results of eight population screenings testing within-population preference (assay 1 in Fig. 1, *n* = 16). Insets show results of eight within population dual-choice assays using clones of most-preferred and least preferred accessions from each population (assay 2 in Fig. 1, *n* = 8). Test statistics are provided in Table 1. The boxes surrounding accession names indicate the accessions used in assay 2. Dashed lines indicate damage distribution when slugs would have equally preferred all accessions tested

**Fig. 3:**
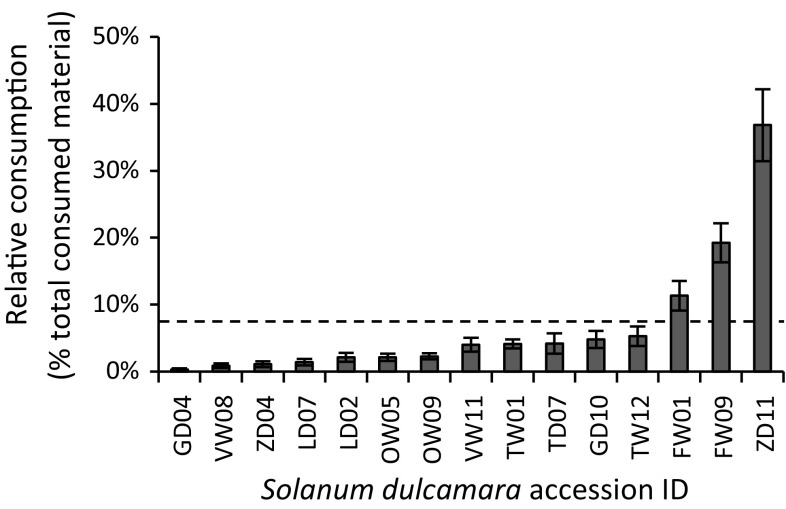
Mean relative consumption (± SE) of grey field slugs on leaf discs of 15 *Solanum dulcamara* accessions characterized by contrasting feeding preference in within population comparisons (assays 1 and 2 in Fig. 1) when offered simultaneously (assay 3 in Fig. 1). Friedman test for overall preference: *n* = 16, *df* = 14, *χ*^2^ = 109.09, *P* ≤ 0.001. Codes of *S. dulcamara* accessions as in Fig. 2. Dashed line indicates damage distribution when slugs would have equally preferred all accessions tested

Slugs showed significant variation in feeding preferences in all eight independent population screenings (assay 1, Fig. 2, Friedman test Table 1). Differences in the relative leaf disc consumption between the most and least preferred accessions within populations ranged from 8% in Texel Dry (TD07: 13%; TD11: 5%) to 62% in Zandvoort Dry (ZD11: 63%; ZD04: 1%). Pair-wise assays with the most- and least-preferred accessions from each population (assay 2) showed that the preference ranking remained consistent when using vegetative clones of the original plant (insets Fig. 2, Wilcoxon test Table 1). In this second assay the difference in relative consumption between the two accessions was lowest for Limburg Dry (28%) and highest in Zandvoort Dry (89%), which illustrates a particularly strong difference in slug preference for accessions from the latter population. Significant differences in slug preference were also observed when all accessions were offered simultaneously (assay 3, Fig. 3). The relative ranking between the most- and least-preferred accessions of each population remained largely the same. Note, however, that due to variation in overall palatability among populations, some of the accessions that were highly preferred in the within-population screenings (such as LD07 and OW05) appeared to be among the least preferred accessions in this overall assay, and vice versa (FW01). Relative leaf disc consumption did not correlate with specific leaf area (Pearson’s *r* = 0.07, *P* = 0.81).

**Table 1:** Test statistics on relative leaf disc consumption by the grey field slug (*D. reticulatum*) in two independent preference assays

Population	Friedman test (assay 1)			Wilcoxon test (assay 2)		
	*n*	*df*	*χ* ^2^	*n*	Ties	*V*
TD	16	11	28.93**	8	1	28.0*
TW	16	11	89.06***	8	0	33.0*
FW	16	11	28.65**	8	1	28.0*
ZD	16	11	57.29***	8	0	36.0*
OW	16	11	82.75***	8	0	36.0*
VW	16	11	103.54***	8	0	36.0*
GD	16	11	26.94**	8	0	36.0*
LD	16	10	22.37*	8	1	28.0*

Friedman rank sum test statistics show the results for within-population preference assay (assay 1 in Fig. 1) and Wilcoxon signed-rank test results for within-population paired dual preference assay using the most- and least-preferred accessions from assay 1 (assay 2 in Fig. 1)

***P ≤ 0.001, **P ≤ 0.01, *P ≤ 0.05

### Chemical leaf profiles and their relation with slug preference

**Fig. 4:**
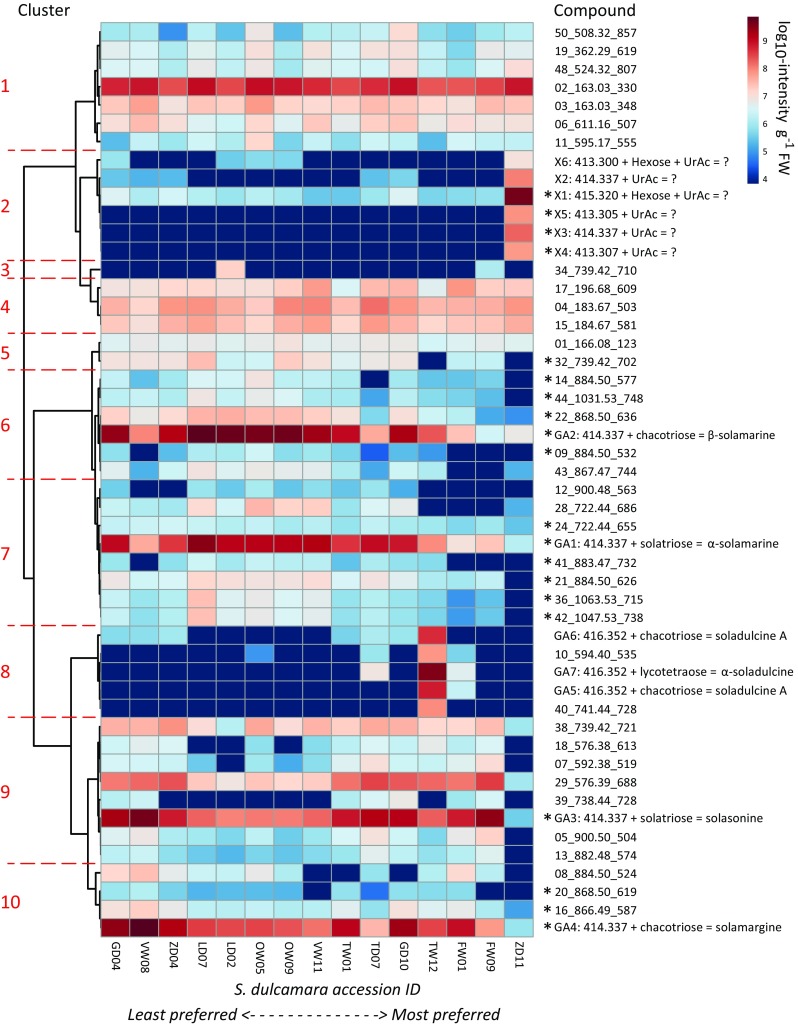
Abundance of 50 most prominent compounds in single samples of 15 *Solanum dulcamara* accessions which were used in a full choice preference assay (assay 3 in Fig. 1). Accessions are arranged from least preferred (left) to most preferred (right). Unidentified compounds are indicated by a compound number, the quantified ion mass (m/z) and its retention time (sec). A selection of 13 compounds (GA1-7, X1-6) was putatively identified to have a steroidal aglycon backbone (m/z [M + H]) + glycoside or uronic acid (UrAc) conjugate and given a putative identity (ESM Table 3). The ID of compounds with significant correlations (FDR-corrected *P* < 0.05) with feeding preference of the grey field slug are preceded by an asterisk. Compounds were grouped in clusters based on mutual Pearson correlation (indicated and separated by red dashed lines and numbers in red on the left). The numbers of the individual *S. dulcamara* accessions are preceded by their population code (Fig. 1) and ordered by their rank in assay 3 (Fig. 3)

**Fig. 5:**
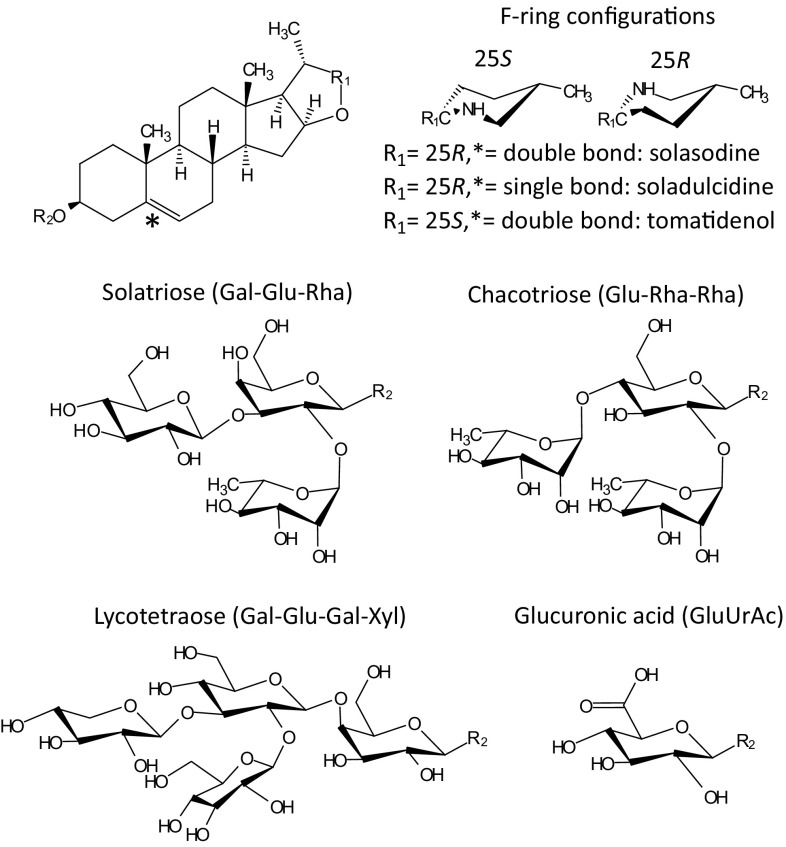
Chemical structure of glycoalkaloid aglycons and their glycoside moieties found in *Solanum dulcamara* using LC-qToF-MS. The configuration of the F-ring on position R_1_ and the saturation level of the C5-C6 bond determine the type of aglycon backbone, which is conjugated to a glycoside moiety on position R_2_

The abundance of the 50 most prominent compounds found in the *S. dulcamara* leaf samples illustrate the chemical diversity among the 15 accessions (Fig. 4). Based on mutual Pearson correlations we were able to distinguish 10 clusters. Correlation analyses of the mean relative consumption of GFS (assay 3) with the 50 most prominent compounds in the metabolic profiles of the accessions used in assay 3 revealed 20 compounds which were significantly correlated with slug preference (FDR-adjusted *P* value < 0.05 as summarised in ESM Table 2). The compounds that correlated with slug preference were found in five clusters; those in clusters 6, 7, 9 and 10 were negatively correlated with slug preference and the compounds in cluster 2 were positively correlated with slug preference. The chemical structure of prominent compounds representing the clusters was further evaluated (GA1-4 from cluster 6, 7, 9 and 10, GA5-7 from cluster 8 and X1-6 from cluster 2, ESM Table 3). All four prominent compounds from clusters that negatively correlated with slug preference were (putatively) identified as structurally related glycosylated steroidal alkaloids (Fig. 5). Based on their mass spectra and co-elution with reference standards, GA3 (cluster 9) and GA4 (cluster 10) were identified as the solasodine-type glycoalkaloids (GAs) solasonine and solamargine, respectively. GA1 and GA2 were tentatively identified as tomatidenol-type glycoalkaloids which are conjugated with different glycoside moieties (Fig. 5).

GA1-4 were the dominant compounds in all accessions but TW12 and ZD11 (Fig. 4). The four GAs occurred in relatively equal ratios in GD04, ZD04, TW01, and GD10. In accessions LD02, LD07, OW05, OW09, and VW11, the tomatidenol-type GAs (GA1-2) were the most prominent, and in two, VW08 and FW01, the solasodine-type GAs (GA3 and 4) dominated the chemical profile (Fig. 4). FW09 mainly contained a single GA, namely GA3 (solasonine) and TD07 mainly contained GA1 and GA3. Accessions TW12 and ZD11 were found to have particularly deviant chemical profiles compared to the other accessions. TW12 was the only accession with a high level of soladulcidine/tomatidine-type GAs (cluster 8, GA5-7 in Fig. 4). Additionally, this accession contained intermediate levels of the two tomatidenol- and two solasodine-type GAs 1–4. Interestingly, the highly preferred accession ZD11 only contained minor levels of the common GAs (GA1–4) found in the other accessions. Instead, it contained mainly saponins (X1, 4–6, Fig. 4) as well as GAs (X2 and 3), which were all conjugated with glucuronic acid instead of the more common combinations of monosaccharides (Fig. 5, ESM Table 3).

## Discussion

Our study revealed significant constitutive variation in plant resistance to the slug *D. reticulatum* within and among eight wild *S. dulcamara* populations from the Netherlands. By utilizing a metabolomics approach to analyse the underlying chemical mechanisms, we identified four prominent steroidal glycoalkaloids (GAs) showing particularly strong negative correlations with slug feeding preference. This is in line with previous studies reporting toxic or repellent effects of different types of alkaloidal secondary metabolites to gastropods (Aguiar and Wink 2005; Bog et al. 2017; Speiser et al. 1992; Wink 1984) and insect herbivores (Altesor et al. 2014; Hare 1983).

In addition, we found considerable chemotypic diversity in GA composition among accessions. The consistency of GFS preference for accessions, as tested using clones of the original seedling in three sequential assays, suggests that the chemical composition of GAs is genetically determined (Willuhn 1966). Moreover, this also suggests that overall slug preference or relative GA levels were not affected by environmental factors, such as seasonal differences (Hare 1983), when plants are grown under regulated greenhouse conditions. It did not seem to matter which of the common *S. dulcamara* GAs dominated the profile; plants with either solasodine-based (GA3, GA4) or tomatidenol-based (GA1, GA2) alkaloids as the main GAs were equally resistant to slug feeding. Moreover, TW12, which contained at least three additional GAs of the soladulcidine/tomatidine type (GA5-7), was not significantly more or less preferred than, for example, accession GD10, which only contained GA1-4. This indicates that the different classes of GAs do not show synergistic effects on slug preference as previously reported for snails feeding on potato (Smith et al. 2001). It thus seems likely that differences in total GA-concentration in the leaves were underlying the observed variation in feeding preferences, rather than GA structural diversity per se. Additional experiments that specifically manipulate GA composition, for example gene editing technologies such as CRISPR/Cas9, are needed to establish a firm correlation between variation in GA composition and slug resistance.

Previous studies found β-solamarine, solasonine and solamargine from various wild *Solanum* species to be lethal to aquatic snails when administered to the water (Alzerreca and Hart 1982; Njeh et al. 2016; Wanyonyi et al. 2002). When ingested, GAs affect neurotransmitters and additionally disrupt cell function by complexation with sterols in the cell membrane (Milner et al. 2011; Moses et al. 2014; Roddick et al. 2001). However, gastropods may also be able to endure toxic substances. Some gastropod species have been shown to possess effective microsomal detoxification mechanisms to cope with alkaloids to a certain extent (Aguiar and Wink 2005). The same authors suggested that cytochrome P450 oxidizing enzymes play a central role (Aguiar and Wink 2005). However, further experimental testing of GA metabolism, for example by feeding labelled GAs to slugs, is necessary to support this hypothesis. We did not explicitly test for potential toxic effects in our study; this would require longer term performance assays including measurements of slug survival. In our assays, the GAs were likely serving as deterrents due to the bitter taste that GAs may cause, as evidenced by the common name of *S. dulcamara;* Bittersweet nightshade.

In addition to the different GA chemotypes which have been described in *S. dulcamara* previously (Eich 2008; Mathé 1970; Willuhn 1966), we also found a hitherto undiscovered chemotype which basically lacked the typical *S. dulcamara* GAs. The most preferred accession, ZD11, appeared to possess a novel type of GA, consisting of a common GA aglycon conjugated with uronic acid (ESM Table 2). Whereas glucuronic acid conjugates of triterpenoid saponins have been reported before in the congeneric *Solanum lyratum* (Sun et al. 2006; Yahara et al. 1985), we found no records in the literature that similar conjugates, as found in accession ZD11, have been reported for GAs (Eich 2008). Seen the close structural similarity and biosynthetic relationships between saponins and GAs, it is not improbable that these glucuronic conjugates might co-occur in a single plant species. In eggplant (*Solanum melongena*) it was found that two similar, though separate, glucosyltransferases with a low substrate specificity were responsible for the 3-O-glucosylation of steroidal saponins as well as GAs (Paczkowski et al. 1998). *S. dulcamara* likely has similarly unspecific glycosyltransferases, which makes it plausible that we would find both saponins and GAs conjugated to glucuronic acid in *S. dulcamara*. Further studies comparing the genomes or transcriptomes of ZD11 with those of the other accessions may reveal the underlying differences in biosynthetic genes (see for example Itkin et al. 2013).

Triterpenoid saponins, such as diosgenin, are not only structurally closely related to GAs, but may also serve similar functions in protecting the plant against herbivores and pathogens (Eich 2008). In *Barbarea vulgaris*, for instance, saponins confer resistance to specialist flea beetles, which are not affected by glucosinolates, the typical defences in *B. vulgaris* and other Brassicaceae (Kuzina et al. 2009). Given the fact that the insect herbivore community of *S. dulcamara* is dominated by several specialist (flea) beetle species (Calf and van Dam 2012; Lortzing et al. 2016; Viswanathan et al. 2005), it is very well possible that the loss of resistance to slug feeding in ZD11 is traded-off by an increased resistance to beetle feeding. Moreover, the source population of ZD11 is located in the dry coastal sand dunes of the Dutch western coast (Fig. 1). In this environment slug feeding may be less frequent, thus providing a window of opportunity for these chemotypes to survive and propagate in this particular population. Our recent finding of another individual with the same chemotype in the same seed batch as ZD11 seems to point in this direction (data not presented). However, an assessment of the local gastropod and insect abundance in combination with transplantation experiments would be needed to unequivocally assess whether herbivore community composition may play a role in the selection for specific chemotypes.

Our results also stress the role and importance of the glycosylation of bioactive molecules, such as GAs. In potato (*Solanum tuberosum*), for instance, the feeding inhibitory effect of chacotriose conjugates on snails was found to be stronger than that of solatriose conjugates (Smith et al. 2001). Another example of the importance of glycosylation comes from the Colorado potato beetle (*Leptinotarsa decemlineata*). This specialist beetle which feeds on a wide range of solanaceaous plants also uses *S. dulcamara* as a wild host in Europe and the USA (Calf and van Dam 2012; Hare 1983). However, it did not feed on species that contain high levels of tetraose conjugates as was found in a comparison of six resistant wild *Solanum* species (Tai et al. 2014). This would lead to the hypotheses that accession TW12, the only accession possessing a tetraose side chain (ESM Table 3), may be more resistant to these beetles than the others.

Our assays revealed high levels of intraspecific variation in slug-resistance within populations of *S. dulcamara*, with 2–60 fold variation in preference for accessions in a population. This suggests that gastropods may impose strong selection on defence traits in natural populations by choosing among the different chemotypes present in a population. This may eventually lead to locally adapted populations, particularly when gastropods are abundant (Kalske et al. 2012; Laine 2009; Scriber 2002). The results of the full choice comparison of all selected accessions (Fig. 3) also suggest that there may be a degree of population differentiation, as some populations overall were more preferred by slugs than others. However, this difference did not appear to be linked to the local abiotic conditions at the sites where seeds for this study were collected. For example, local hydrological conditions both in the FW and OW populations are likely favouring gastropod abundance and should be favouring selection of resistant genotypes. However, on average FW accessions were considerably more preferred by GFS when given the choice than accessions from other populations, indicating that other factors may contribute to chemical population characteristics than habitat type.

In conclusion, plants may employ different strategies and different combinations of secondary plant compounds to reduce herbivore damage. Intraspecific variation in resistance is the basis for the evolution of herbivore resistance traits. We found that *S. dulcamara* shows significant variation in slug resistance, which was closely linked to differences in their chemical profiles, especially that of GAs. This does not preclude that other defences known to be present and to vary in *S. dulcamara*, such as polyphenoloxidases, peroxidases, protease inhibitors and extrafloral nectar (Lortzing et al. 2016; Nguyen et al. 2016; Viswanathan et al. 2008), play an additional role in slug resistance. We argue that slugs, in addition to insects and pathogens, thus may exert a strong selection pressure on the chemical profiles of plants. This may be especially so during seedling establishment, a stage which had been shown to be exceptionally vulnerable to slug herbivory (Smith et al. 2001). Therefore, slugs and the damage they do to plants should be more often considered when studying the ecological roles and evolutionary origins of chemical variation in plants.

## Electronic supplementary material

Below is the link to the electronic supplementary material.

Supplementary material 1 (XLSX 29 kb)
